# Positive feedback loop between MAPK and aquaporin 7 regulates autophagy and apoptosis induced by palmitate in RIN‐m5f cells

**DOI:** 10.1002/2211-5463.70011

**Published:** 2025-03-24

**Authors:** Maoqi Wang, Jiang Tan, Xueting He, Yuqin Chen, Guoping Qiu, Mei Yang

**Affiliations:** ^1^ Department of Anatomy, Institute of Neuroscience, College of Basic Medicine Chongqing Medical University China; ^2^ Institute of Life Science Chongqing Medical University China

**Keywords:** apoptosis, aquaporin 7, autophagy, MAPK, palmitate

## Abstract

Type 2 diabetes mellitus (T2DM) is characterized by peripheral blood insulin resistance and progressive pancreatic β‐cell dysfunction which is closely related to apoptosis of β‐cells. Aquaporin 7 (AQP7) is the only aquaglyceroporin protein expressed in pancreatic β‐cells. However, the relationship between AQP7 and autophagy remains unexplored, with limited studies investigating its link to islet β‐cell apoptosis. In our study, we utilized an *in vitro* model involving palmitate‐treated rat pancreatic β‐cells (RIN‐m5f) to examine these relationships. Our aim was to investigate the effects of AQP7 on autophagy and apoptosis by examining LC3 lipidation levels and p62 expression in pancreatic islet β‐cells, thereby elucidating potential underlying mechanisms. Our results showed that phosphorylation of p38 and c‐Jun‐terminal kinase (JNK) increased in response to palmitate treatment, indicating the activation of these signaling pathways. Conversely, AQP7 expression decreased, reduced autophagy, and promoted apoptosis. AQP7 knockdown activated the p38 and JNK signaling pathways, inhibited autophagy (as evidenced by LC3 lipidation and p62 expression), and increased apoptosis. Furthermore, AQP7 overexpression repressed palmitate‐induced apoptosis and alleviated autophagy inhibition by suppressing the p38 and JNK mitogen‐activated protein kinase (MAPK) signaling pathways. Our results suggest a positive feedback loop between MAPK signaling and AQP7 that regulates autophagy and apoptosis in RIN‐m5f cells under high‐fat conditions.

## Graphical abstract

T2DM is characterized by peripheral blood insulin resistance and progressive pancreatic β‐cell dysfunction. Here, we showed that AQP7, the only aquaglyceroporin protein, regulates autophagy and apoptosis in pancreatic β‐cells. We propose the existence of a positive feedback loop between MAPK signaling and AQP7 that regulates autophagy and apoptosis in RIN‐m5f cells under high‐fat conditions.

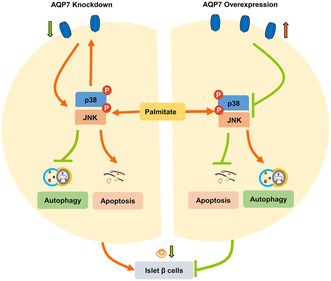

**Abbreviations**
ANOVA: analysis was performed using Student's *t*‐test or one‐way analysis of variance
AQP7: aquaporin 7 caspase 3 cysteinyl aspartate‐specific proteinase
ERK1/2: extracellular signal‐regulated kinases 1/2
JNK: Jun N‐terminal kinase
MAP1LC3: LC3 microtubule‐associated protein 1A/1B‐light chain 3
MAPK: mitogen‐activated protein kinase
p62: p62/SQSTM1, sequestosome‐1
PA: palmitic acid
PBST: phosphate‐buffered saline with tween
T2DM: type 2 diabetes mellitus

Diabetes mellitus (DM) is a significant global health concern. Type 2 diabetes mellitus (T2DM), also known as non‐insulin‐dependent diabetes, is the most prevalent type of diabetes and accounts for more than 90% of all diabetes cases [1]. It is characterized by peripheral insulin resistance and progressive pancreatic β‐cell dysfunction, closely linked to β‐cell apoptosis [2, 3]. Patients with T2DM are also often obese or overweight. In individuals with obesity, there is typically an elevation in free fatty acids within expanded adipose tissue [4]. Palmitic acid (PA), a type of free fatty acid, is the primary cause of islet β‐cell lipotoxicity and apoptosis [5]. Meanwhile, palmitate also enhances basal and glucose‐stimulated insulin secretion in β‐cells, with chronic exposure ultimately leading to β‐cell dysfunction [6, 7, 8]. Cellular metabolism involves both anabolism and catabolism, intracellular autophagy playing a crucial role in recycling organelles and proteins [9]. Palmitate (PA) can block autophagic flux by impairing lysosome‐to‐autophagosome conversion, resulting in apoptosis across human islets and pancreatic β‐cell lines [10, 11].

Aquaporins comprise a family of 13 members (AQP0–12) that transport water across cell membranes. They are categorized into two subfamilies based on their transport functions: aquaporins permeable to water (AQP0, AQP1, AQP2, AQP4, AQP5, AQP6, and AQP8) and aquaglyceroporins (AQP3, AQP7, AQP9, AQP10, and AQP11), which are permeable to small solutes like glycerol and urea, alongside water. Additionally, there are unorthodox aquaporins (which have little conserved amino acid sequences around the asparagine‐proline‐alanine (NPA) boxes) or superaquaporins (AQP11 and AQP12). Aquaglyceroporins play important roles in the different organs in which they are expressed, including the liver, adipose tissue, and pancreas, where they are involved in energy metabolism [12, 13]. AQP7 is the only aquaglyceroporins channel expressed in pancreatic β‐cells [14], and its deficiency leads to patients with obesity [15]. AQP7 is expressed in the β‐cells in the islets of Langerhans of both rats and mice. AQP7 is localized in the cell membrane of pancreatic β‐cells [16, 17, 18, 19]. AQP7 regulates triacylglycerol synthesis, controls glycerol levels in pancreatic islets, and influences insulin secretion and pancreatic β‐cell proliferation [6, 17, 20]. Apoptosis‐related gene expression is associated with AQP7 in pancreatic islets of AQP7 knockout mice [14]. Moreover, AQP7 deficiency in cardiomyocyte has been linked to increased apoptosis [21]. However, the relationship between AQP7 and autophagy remains unexplored and few studies have evidenced its association with islet β‐cell apoptosis. In our previous study, we demonstrated that high glucose and high fat downregulate AQP7, and metformin rescues AQP7 expression to enhance insulin secretion. Nonetheless, the specific mechanism underlying the role of AQP7 in autophagy and apoptosis remains unclear [22].

Mitogen‐activated protein kinase (MAPK) signaling involves a cascade of events that regulate different biological processes associated with the pathogenesis of obesity‐induced insulin resistance and T2DM [23]. Key constituents of this pathway include c‐Jun N‐terminal kinase (JNK), extracellular signal‐regulated kinases 1/2 (ERK1/2), and p38 kinase (p38) [24]. Numerous studies have underscored the pivotal role of MAPK signaling in regulating cellular dysfunction, particularly its association with autophagy and apoptosis [25, 26, 27]. Our previous study confirmed that MAPK signaling regulates the expression of AQP7 [13]. However, whether AQP7 influences MAPK signaling also remains unknown. Thus, the present study aimed to investigate whether AQP7 impacts autophagy and apoptosis via MAPK signaling in pancreatic islet β‐cells.

## Materials and methods

### Cell culture

RIN‐m5f cells (1101RAT‐PUMC000386) were obtained from the Cell Resource Center, IBMS, CAMS/PUMC (Beijing, China). Cells were cultured in RPMI 1640 (Gibco, Grand Island, NY, USA), supplemented with 10% fetal bovine serum (Foundation B™ Fetal Bovine Serum‐Item: 900‐208; Gemini Bio Company, West Sacramento, CA, USA) and 50 U·mL^−1^ penicillin–streptomycin at 37 °C in a humidified atmosphere with 5% CO_2_. Both the palmitate‐treated and solvent control groups underwent simultaneous treatment for 24 h. The cells in the treatment group were incubated in the presence of palmitate (0.25 mm). Before being treated with 0.25 mm palmitate, cells were pretreated for 1 h with the JNK inhibitor SP600125 (10 μm) or the p38 MAPK inhibitor SB203580 (10 μm) or for 2 h with anisomycin alone (a strong agonist of p38 and JNK; 2 μm).

### Western blot analysis

RIN‐m5f cells were homogenized in RIPA lysis buffer (Beyotime Institute of Biotechnology, Shanghai, China) containing a protease and phosphatase inhibitor cocktail in lysis buffer. Assays for the homogenate protein content were conducted using a BCA protein assay kit (Beyotime Institute of Biotechnology). Fifty micrograms of extracted proteins were separated using 10% or 12.5% SDS/PAGE before being applied to membranes. Proteins phosphorylated on blotted membranes were blocked with bovine serum albumin (5%), whereas non‐phosphorylated proteins were blocked with skim milk (5%). Membranes (PVDF 0.22 μm; MerckMillipore, St. Louis, MI, USA) were first blocked and then incubated at 4 °C with primary antibodies, including AQP7 antibody (SC376407, 1 : 200, mouse anti‐AQP7; Santa Cruz, CA, USA), p38 (66234‐1‐lg, 1 : 1000, mouse anti‐p38; Proteintech, Wuhan, China), phosphorylated p38 (#4511, 1 : 1000, rabbit anti‐p‐p38; Cell Signaling Technology, Boston, MA, USA), ERK1/2 (67170‐1‐Ig, 1 : 1000, mouse anti‐ERK; Proteintech), phosphorylated ERK1/2 (#4370, 1 : 1000, rabbit anti‐p‐ERK1/2; Cell Signaling Technology), JNK (66210‐1‐lg, 1 : 1000, mouse anti‐JNK; Proteintech), phosphorylated JNK (#4668, 1 : 1000, rabbit anti‐p‐JNK; Cell Signaling Technology), α‐tubulin (66031‐1‐lg, 1 : 5000, mouse anti‐α‐tubulin; Proteintech), GAPDH (60004‐1‐lg, 1 : 5000, mouse anti‐GAPDH; Proteintech), LC3B (A19665, 1 : 1000, rabbit anti‐LC3B; Abclonal, Wuhan, China), p62 (A19700, 1 : 1000, rabbit anti‐p62; Abclonal) or caspase3 (AF6311, 1 : 1000, rabbit anti‐caspase3; Affinity Biosciences, Cincinnati, OH, USA). Subsequently, the membranes were treated with secondary antibodies conjugated to horseradish peroxidase (goat anti‐rabbit HRP or goat anti‐mouse HRP; ZSGB Bio, Beijing, China). The specific reactions were visualized using a chemiluminescent substrate (ECL), and quantity one software (Bio‐Rad, Hercules, CA, USA) was used to densitometrically quantify the protein bands. The results were expressed ratios such as AQP7/α‐tubulin, p‐MAPK/MAPK, LC3II/LC3I, p62/GAPDH, and caspase3/α‐tubulin. Each measurement was performed in triplicate.

### Flow cytometry analysis

Flow cytometry was performed as previously described. Apoptotic cells were detected using an annexin V‐fluorescein isothiocyanate/propidium iodide apoptosis detection kit (BD Biosciences, Franklin Lakes, NJ, USA). The combined percentage of early and late apoptotic cells was used to determine the overall percentage of apoptotic cell.

### Hoechst33258 staining

Hoechst 33258 is a fluorescent stain for labeling DNA in fluorescence microscopy and used for apoptosis detection. For apoptosis detection, sections were treated with Hoechst 33258 (C1018; Beyotime, Beijing, China) for 5 min at room temperature, followed by three washes with PBST (1% PBS with tween). Fields of view (400×) were randomly captured under a fluorescence microscope (DM2500; Leica, Wetzlar, Germany), with each group repeated three times [28].

### Lentivirus infection

To achieve stable overexpression of AQP7 in RIN‐m5f cells, lentiviruses encoding the full‐length rat AQP7 gene under Ubi‐AQP7‐MCS‐3FLAG‐SV40‐EGFP‐IRES‐puromycin construct, along with an empty vector control Ubi‐MCS‐3FLAG‐SV40‐EGFP‐IRES‐puromycin, were transfected into RIN‐m5f cells. AQP7 overexpressing clones were selected 2 weeks post‐transfection using puromycin at concentrations ranging from 0.5 to 2 μg·mL^−1^. For AQP7 knockdown, lentivirus carrying hU6‐AQP7‐MCS‐CBh‐gcGFP‐IRES‐puromycin with target sequences (5′‐CTGCAGCTACCAC CTACTTAA‐3′) were employed, while hU6‐MCS‐CBh‐gcGFP‐IERS‐puromycin sequences (5′‐TTCTCCGAACGTGTCACGT‐3′) served as the negative control. Knockdown or overexpression efficiency was assessed via western blotting. Virus packaging, vector construction (verified by Sanger sequencing), and collection of the matching viral supernatants were carried out by GeneChem Co., Ltd. (Shanghai, China).

### Statistical analysis

All data are presented as mean ± SEM, and statistical analysis was performed using Student's *t*‐test or one‐way analysis of variance (ANOVA) with graphpad prism v8.0 software (Boston, MA, USA). *P* value < 0.05 was considered statistically significant. Each experiment was conducted three times, with batches of cells used for each experiment.

## Results

### Palmitate reduced AQP7 expression, activated p38 and JNK signaling pathways, inhibited autophagy, and promoted apoptosis

**Fig. 1:**
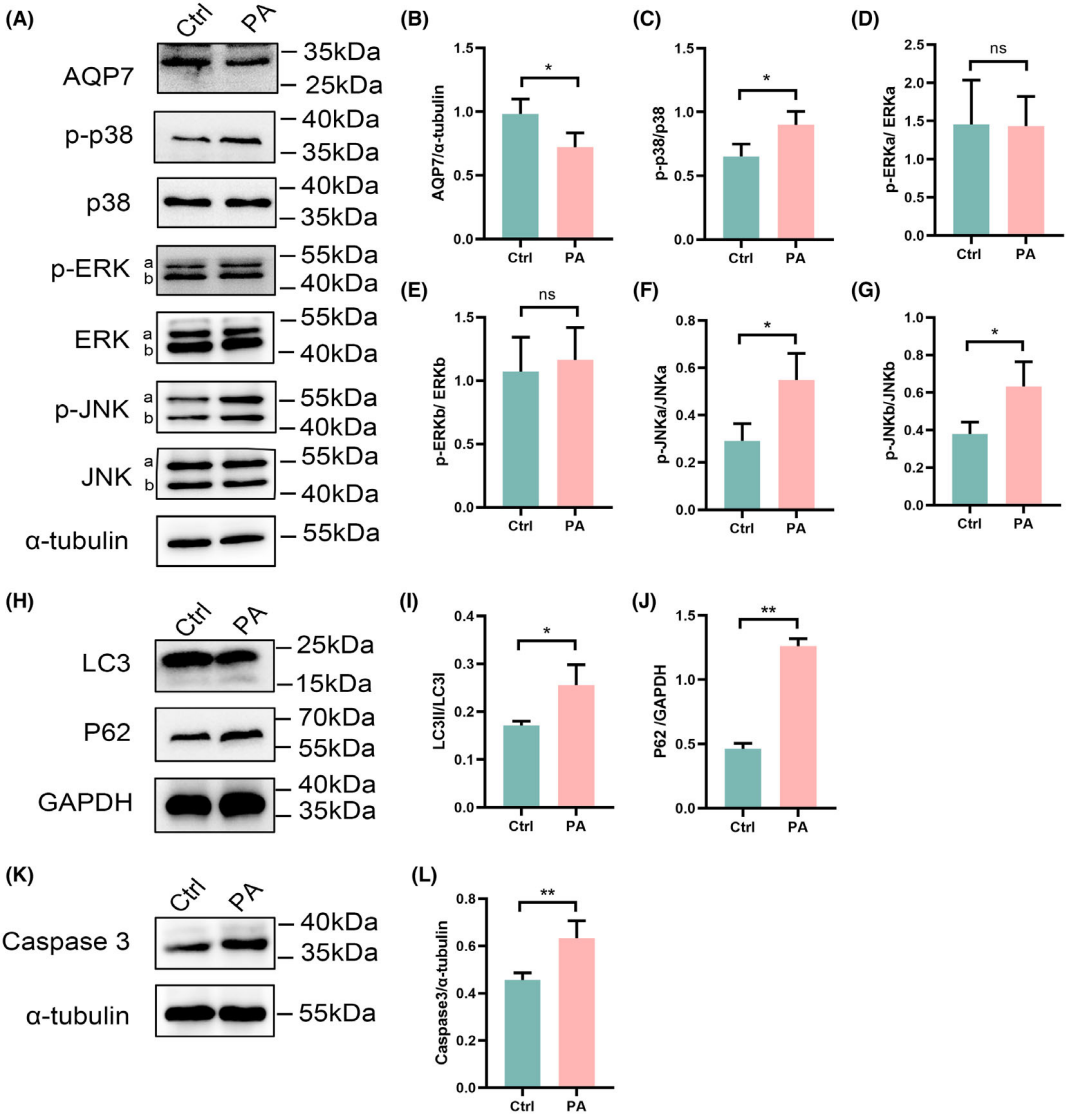
Effect of palmitate exposure on AQP7, autophagy, apoptosis, and MAPK expression in RIN‐m5f cells, detected via western blot analysis. (A) Protein expression of AQP7, p‐p38, p38, p‐ERK, ERK, p‐JNK and JNK in RIN‐m5f cells. (B) Ratio of AQP7/α‐tubulin in RIN‐m5f cells. (C) Ratio of p‐p38/p38 in RIN‐m5f cells. (D, E) Ratio of p‐ERK/ERK in RIN‐m5f cells. (F, G) Ratio of p‐JNK/JNK in RIN‐m5f cells. (H) Expression of LC3 and p62 proteins in RIN‐m5f cells. (I) Ratio of LC3II/LC3I in RIN‐m5f cells. (J) Ratio of p62/GAPDH in RIN‐m5f cells. (K) Protein expression of caspase 3 in RIN‐m5f cells. (L) Ratio of caspase 3 to α‐tubulin in RIN‐m5f cells. Results are expressed as mean ± SEM of three independent experiments. Experimental groups include control group (Ctrl), and 0.25 mm palmitate group (PA). Statistical significance indicated as: ns, not significant; *P < 0.05; **P < 0.01, determined by unpaired two‐tailed Student's *t*‐test (B–L).

**Fig. 2:**
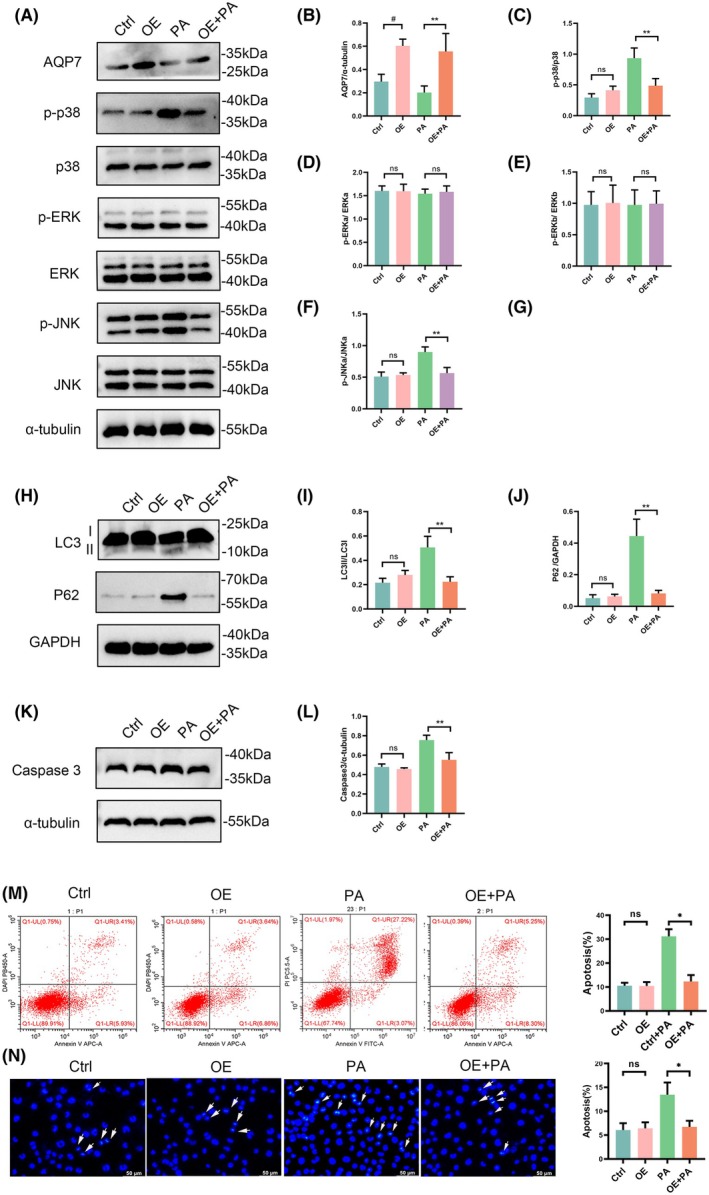
Effect of AQP7 overexpression (OE) on MAPK expression, autophagy, and apoptosis in RIN‐m5f cells. (A) Protein expression of AQP7, p‐p38, p38, p‐ERK, ERK, p‐JNK, and JNK in RIN‐m5f cells following AQP7 overexpression. (B) Ratio of AQP7/α‐tubulin in RIN‐m5f cells. (C) Ratio of p‐p38/p38 in RIN‐m5f cells. (D, E) Ratio of p‐ERK/ERK in RIN‐m5f cells. (F, G) Ratio of p‐JNK/JNK in RIN‐M5f cells. (H) Protein expression of LC3 and P62 in RIN‐m5f cells after AQP7 overexpression. (I) Ratio of LC3II/LC3I in RIN‐m5f cells. (J) Ratio of p62/GAPDH in RIN‐m5f cells. (K) Protein expression of caspase 3 in RIN‐m5f cells. (L) Ratio of caspase 3/α‐tubulin in RIN‐m5f cells. (M) Flow cytometry analysis and (N) Hoechst staining to determine the apoptosis rate after AQP7 overexpression. White arrows indicate apoptotic cells. Results are expressed as mean ± SEM of three independent experiments. Experimental groups include control group (Ctrl), AQP7 overexpression group (OE), control with 0.25 mm palmitate group (PA), AQP7 overexpression with 0.25 mm palmitate group (OE + PA). Statistical significance indicated as: ^#^ *P* < 0.05 vs. Ctrl group; *P < 0.05 vs. Ctrl group; **P < 0.01 vs. Ctrl group; ns, not significant, analyzed using two‐way ANOVA (B–L).

To assess the impact of palmitate on AQP7, the MAPK signaling pathway, autophagy, and apoptosis in RIN‐m5f cells, we examined the expression of AQP7, phosphorylated and total p38, ERK, and JNK MAPK, as well as LC3 (microtubule‐associated protein 1A/1B‐light chain 3, MAP1LC3), p62 (p62/SQSTM1, Sequestosome‐1), and caspase 3 (cysteinyl aspartate‐specific proteinase) using western blotting. Our results showed that palmitate significantly reduced the expression of AQP7 (*P* < 0.05) (Fig. 1A,B). Notably, palmitate triggered significant activation of p38 and JNK MAPK, while ERK levels remained unchanged (Fig. 1A,C–G). Furthermore, an evident increase was observed in the rations of LC3II/LC3I, p62, and caspase 3 in the palmitate‐treated group compared to the control (*P* < 0.05) (Fig. 1H–L). These observations were corroborated by flow cytometry and Hoechst staining, confirming an elevated ratio of apoptosis in RIN‐m5f cells following palmitate treatment (Fig. 2M; Fig. S1).

### Palmitate regulated AQP7 expression, autophagy, and apoptosis through p38 and JNK MAPK signaling pathways

**Fig. 3:**
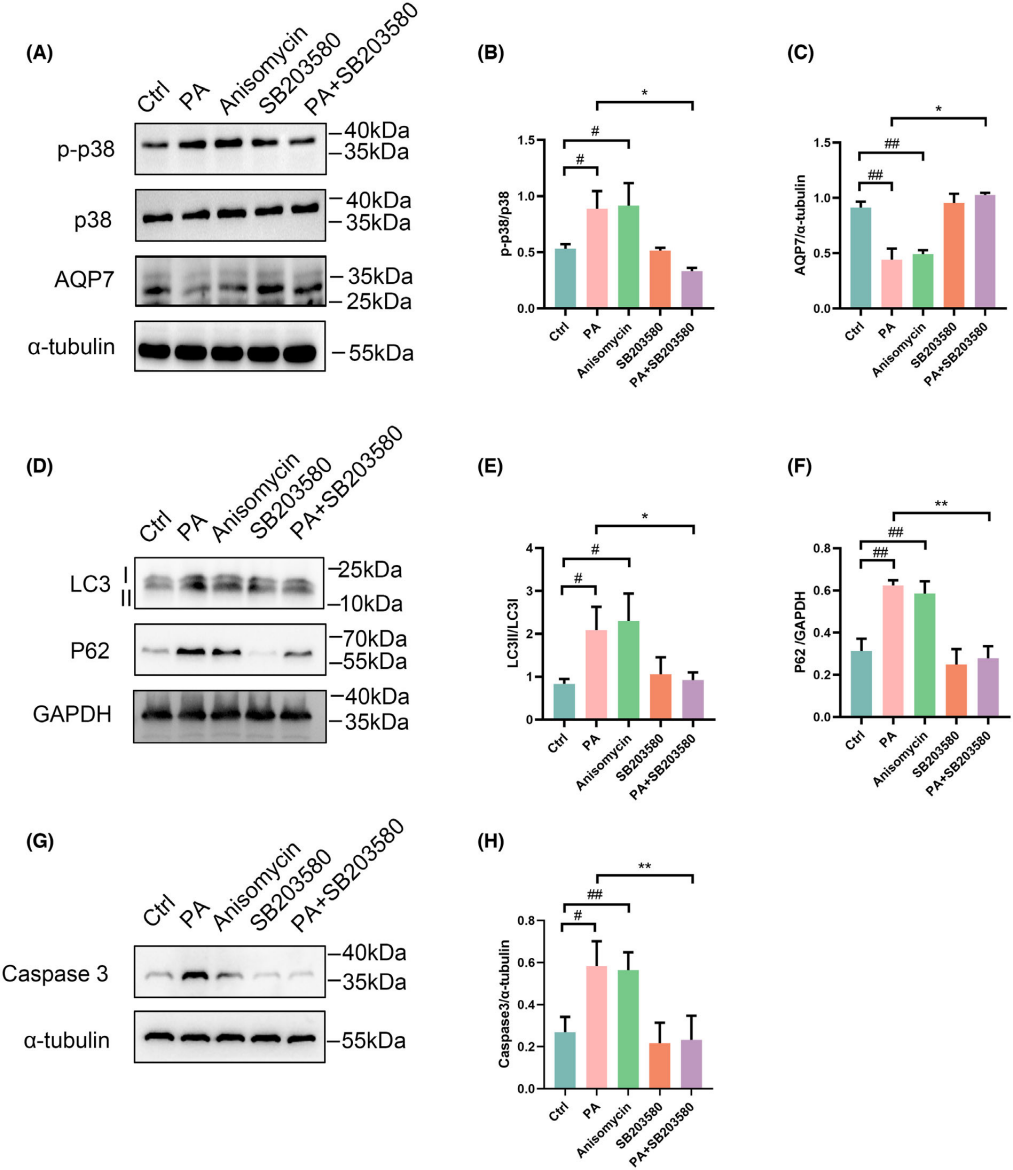
Effects of p38 and JNK MAPK signaling pathway on AQP7, autophagy, and apoptosis in RIN‐m5f cells, detected via western blot analysis. (A) Protein expression levels of p‐p38, p38 and AQP7 in RIN‐m5f cells under various treatments (Ctrl, PA, anisomycin, SB203580, and PA + SB203580). (B) Ratio of p‐p38/p38 in RIN‐m5f cells. (C) Ratio of AQP7/α‐tubulin in RIN‐m5f cells. (D) Protein expression of LC3 and P62 in RIN‐m5f cells. (E) Ratio of LC3II/LC3I in RIN‐m5f cells. (F) Ratio of p62/GAPDH in RIN‐m5f cells. (G) Protein expression of caspase 3 in RIN‐m5f cells. (H) Ratio of caspase 3/α‐tubulin. Results are expressed as mean ± SEM of three independent experiments. Experimental groups include control group (Ctrl), 0.25 mm palmitate group (PA), 2 μm Anisomycin (a potent p38 and JNK agonist) group (Anisomycin), 10 μm SB203580 (a p38 MAPK inhibitor) group (SB203580), and 0.25 mm palmitate and 10 μm SB203580 group (PA + SB203580). Statistical significance indicated as: ^#^ *P* < 0.05 vs. Ctrl group; ^##^ *P* < 0.01 vs. Ctrl group; *P < 0.05 vs. PA group; **P < 0.01 vs. PA group, analyzed using two‐way ANOVA (B–H).

**Fig. 4:**
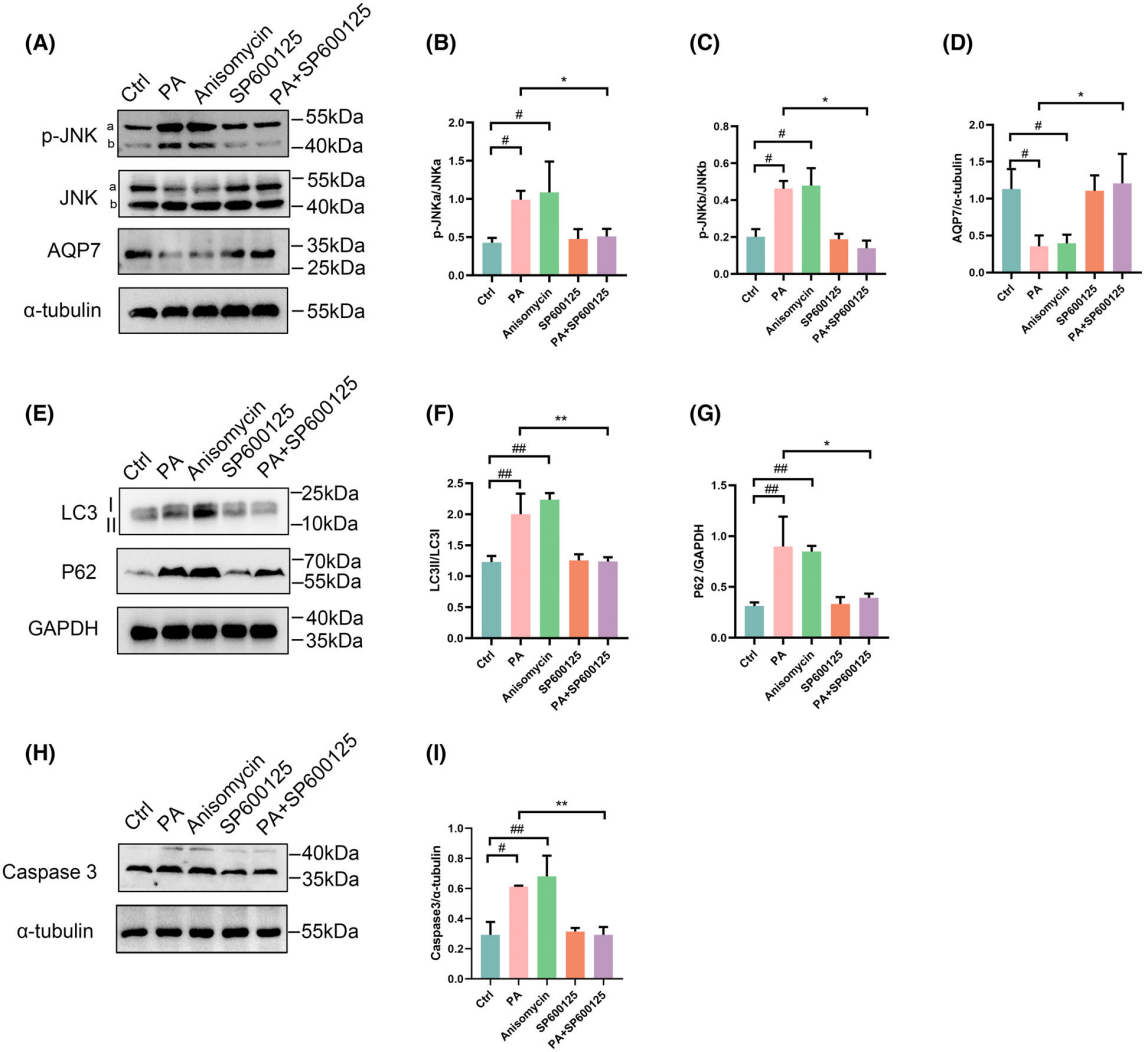
Effects of JNK signaling pathway on AQP7, autophagy, and apoptosis in RIN‐m5f cells, detected via western blot analysis. (A) Protein expression levels of p‐JNK, JNK, and AQP7 in RIN‐m5f cells (Ctrl, PA, anisomycin, SP600125, and PA + SP600125). (B, C) Ratio of p‐JNK/JNK in RIN‐m5f cells. (D) Ratio of AQP7/α‐tubulin in RIN‐m5f cells. (E) Protein expression of LC3 and p62 in RIN‐m5f cells. (F) Ratio of LC3II/LC3I in RIN‐M5f cells. (G) Ratio of p62/GAPDH in RIN‐m5f cells. (H) Protein expression of caspase 3 in RIN‐m5f cells. (I) Ratio of caspase3/α‐tubulin. Results are expressed as mean ± SEM of three independent experiments. Experimental groups include control group (Ctrl), 0.25 mm palmitate group (PA), 2 μm Anisomycin (a potent p38 and JNK agonist) group (Anisomycin), 10 μm SP600125 (a JNK inhibitor) group (SP600125), 0.25 mm palmitate and 10 μm SP600125 group (PA + SP600125). Statistical significance indicated as: ^#^ *P* < 0.05 vs. Ctrl group; ^##^ *P* < 0.05 vs. Ctrl group; *P < 0.05 vs. PA group; **P < 0.01 vs. PA group, determined using two‐way ANOVA (B–I).

We have found that palmitate decreased AQP7 expression and activated p38 and JNK MAPKs. In order to demonstrate that p38 and JNK MAPK signaling pathways are responsible for regulating the expression of AQP7, autophagy and apoptosis, anisomycin (a potent agonist for JNK and p38) was used to activate p38 and JNK MAPK signal pathways. Our results demonstrated that both palmitate and anisomycin significantly activated p38 and JNK signaling while decreasing AQP7 expression (*P* < 0.05) (Figs 3A,B and 4A–C). This suggests that palmitate and anisomycin exert similar effects on AQP7 expression. Moreover, the restoration of AQP7 expression levels with SB203580 (a p38 MAPK inhibitor) (*P* < 0.05) and SP600125 (a JNK inhibitor) following palmitate treatment (Fig. 3A,C), further supports the involvement of p38 and JNK signaling in regulating AQP7 expression induced by palmitate.

Furthermore, our results demonstrated that palmitate increased the ratio of LC3II/LC3I, p62 expression, and caspase 3 expression. To investigate whether palmitate regulates autophagy and apoptosis through p38 and JNK MAPK signaling, we observed a significant increase in LC3II/LC3I, p62 and caspase 3 levels upon activation of p38 and JNK MAPK signaling by anisomycin. Interestingly, inhibition of p38 and JNK signaling with SB203580 and SP600125, respectively, rescued autophagy levels and reduced caspase 3 expression (Figs 3D–H and 4E–I), suggesting that PA regulates autophagy and apoptosis through p38 and JNK MAPK signaling pathways.

### AQP7 knockdown activated p38 and JNK MAPK signaling pathways, inhibited autophagy, and promoted apoptosis

**Fig. 5:**
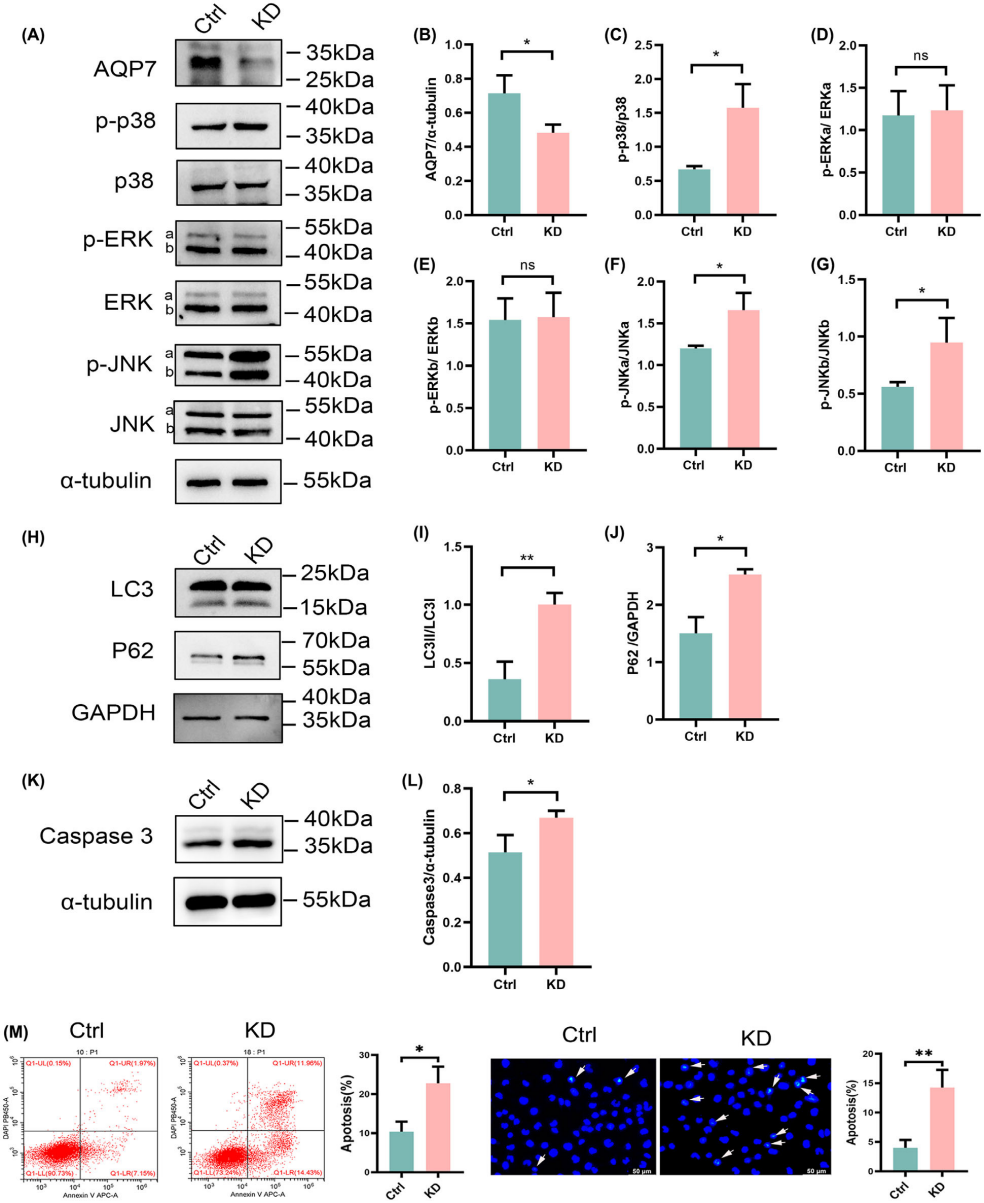
Effect of AQP7 knockdown (KD) on MAPK expression, autophagy, and apoptosis in RIN‐m5f cells. (A) Protein expression of AQP7. (B) Ratio of AQP7/α‐tubulin in RIN‐m5f cells. (A) Protein expression of p‐p38, p38, p‐ERK, ERK, p‐JNK, and JNK in RIN‐m5f cells after AQP7 KD. (C) Ratio of p‐p38/p38 in RIN‐m5f cells. (D, E) Ratio of p‐ERK/ERK in RIN‐m5f cells. (F, G) Ratio of p‐JNK/JNK in RIN‐m5f cells. (H) Protein expression of LC3 and P62 in RIN‐m5f cells after AQP7 KD. (I) Ratio of LC3II/LC3I in RIN‐m5f cells. (J) Ratio of p62/GAPDH in RIN‐m5f cells. (K) Protein expression of caspase 3 in RIN‐m5f cells. (L) Ratio of caspase 3/α‐tubulin in RIN‐m5f cells. (M) Results of flow cytometry analysis and Hoechst staining. White arrows indicate apoptotic cells. Results are expressed as mean ± SEM of three independent experiments. Experimental groups include control group (Ctrl), and AQP7 knockdown group (KD). Statistical significance indicated as: *P < 0.05 vs. Ctrl group; **P < 0.01 vs. Ctrl group; ns, not significant, analyzed using unpaired two‐tailed Student's *t*‐test (B–M).

To assess the impact of AQP7 on MAPK signaling, we established RIN‐m5f cell lines with stable knockdown of AQP7 via lentiviral infection. The efficiency of AQP7 knockdown was determined using western blotting (Fig. 5A). Statistical results showed that AQP7 expression was significantly reduced (*P* < 0.01), with the knockdown efficiency reaching 68%, calculated as the average protein percentage in AQP7‐knocked cells compared to control cell (Fig. 5B). Subsequent analysis showed that p38 and JNK MPAK signaling were activated following AQP7 knockdown, while ERK signaling was not significantly changed (Fig. 5C–G). Subsequently, to investigate whether AQP7 knockdown affected autophagy and apoptosis, we examined the expression of LC3II/LC3I, p62, and caspase 3 following AQP7 knockdown. Our results revealed a notable increase in LC3II/LC3I, p62 expression, and caspase 3 expression unop AQP7 knockdown (Fig. 5H–L). In addition, flow cytometry analysis and Hoechst staining demonstrated that AQP7 knockdown increased apoptosis in RIN‐m5f cells (Fig. 5M).

### AQP7 overexpression rescued autophagy and suppressed palmitate‐induced apoptosis by inhibiting p38 and JNK MAPK signaling pathways

To further elucidate the role of AQP7 in MAPK expression, autophagy, and apoptosis in the presence of palmitate, we established RIN‐m5f cell lines with stable overexpress of AQP7 using lentiviral infection. Western blotting confirmed the effectiveness of AQP7 overexpression at the protein level (Fig. 2A). Statistical analysis also showed that AQP7 expression significantly increased in the OE and OE + PA group (*P* < 0.01) (Fig. 5B). Our results demonstrated that AQP7 overexpression inhibited palmitate‐induced activation of p38 and JNK MAPKs signaling pathways (Fig. 2A,C–G). Additionally, AQP7 overexpression rescued palmitate‐inhibited autophagy and repressed palmitate‐induced apoptosis compared to the control group (Fig. 2H–L). Furthermore, flow cytometry and Hoechst staining demonstrated that AQP7 overexpression inhibited palmitate‐induced apoptosis (Fig. 2M,N).

## Discussion

Obesity, a major risk factor for diabetes, is associated with increased free fatty acid levels. PA, a major component of free fatty acids, has been shown to be associated with apoptosis and dysfunction of islet β function [29, 30]. Autophagy is an important cellular maintenance mechanism that protects the cells from stress and promotes survival. However, impaired autophagy induced by lipid overload in obese individuals can lead to β‐cell dysfunction, with disrupted autophagic flux implicated in lipotoxicity‐induced β‐cell dysfunction [11, 31]. Moreover, autophagy was also found to be inhibited by PA in pancreatic β‐cell lines and primary β‐cells, resulting in the loss of β‐cell function [32]. Nonetheless, the underlying mechanism remains largely unexplored. In present study, we found that palmitate decreased AQP7 expression, increased caspase 3 expression, increased apoptosis, and rised the expression of LC3II/I, P62 to repress autophagy by activating p38 and JNK MAPK signaling in RIN‐m5f cells. Furthermore, AQP7 knockdown activated p38 and JNK kinase signaling, promoted apoptosis, and inhibited autophagy. Conversely, AQP7 overexpression rescued palmitate‐impaired autophagy and inhibited palmitate‐induced apoptosis by suppressing p38 and JNK MAPK signaling. These findings suggest a potential positive feedback loop involving AQP7, p38 and JNK MAPK signaling in regulating autophagy and apoptosis in RIN‐m5f cells. Moreover, we speculate that other signaling pathways may also be involved in the absence of an increase in apoptosis.

Previous studies have demonstrated that AQP7 is the only aquaglyceroporin protein channel present on pancreatic β‐cells, and its inactivation leads to diminished islet and total islet cell mass [14]. Our research team, along with other, has further elucidated the role of AQP7 in insulin secretion, β‐cell mass and various biological functions such as proliferation, adhesion, migration, and membrane permeability of pancreatic β‐cells [22, 33, 34]. Moreover, the human AQP7 gene is also associated with T2DM [35]. These studies suggest that AQP7 is closely linked with T2DM and the function of pancreatic islet β‐cells. Some studies have reported a reduction in the expression of proapoptotic (caspase 3 and Bax) in Aqp7^−/−^ mice. However, the association between AQP7 expression in pancreatic β‐cells and autophagy remains unexplored [14]. Based on these findings, we speculated a potential link between AQP7, apoptosis, and autophagy in pancreatic islet β‐cells. The present study demonstrated that palmitate‐induced reduction in AQP7 expression or shRNA downregulation of AQP7promoted apoptosis and inhibited autophagy in RIN‐m5f cells. These findings suggest that palmitate may affect autophagy and apoptosis in RIN‐m5f cells by reducing AQP7 expression.

Cell death is typically mediated by two important cellular processes: autophagy and apoptosis. The regulation of apoptosis and autophagy frequently involves a complex network of signaling pathways. As previously reported, activation of the p38 and JNK MAPK signaling has been linked to islet β‐cell apoptosis [36, 37, 38]. In a study of islet β‐cell autophagy regulation associated with circular RNAs, MAPK signaling was predicted to be highly correlated with the regulation of autophagy [39]. Therefore, we hypothesized that MAPK signaling is closely related to autophagy and apoptosis in pancreatic β‐cells. RIN‐m5f cells are widely used because of their high insulin. To investigate the effect of lipotoxicity on the autophagy and apoptosis of islet β‐cells, we selected RIN‐m5f cells for palmitate treatment. Our results demonstrated that palmitate suppressed autophagy and promoted apoptosis in RIN‐m5f cells. Palmitate also activated p38 and JNK MAPK signaling pathways while inhibiting AQP7 expression in RIN‐m5f cells. Interestingly, similar results were obtained following AQP7 knock down. In addition, overexpression of AQP7 significantly reversed the effects of palmitate on the p38 and JNK MAPK signaling, autophagy, and apoptosis. Thus, our study suggests a potential a positive feedback loop involving p38 and JNK MAPK singling and AQP7 in the regulation of autophagy and apoptosis. However, no significant changes in ERK MAPK signaling were observed in response to palmitate or AQP7 expression. One study reported that treatment with palmitate (0.4 mm) for 48 h did not influence ERK signaling in pancreatic β‐cells [40], while another study found that short‐time palmitate treatment activated ERK signaling [27]. These discrepancies may stem from variations in culture conditions and treatment durations. Nevertheless, the lack of significant changes in ERK signaling upon AQP7 knockdown or overexpression warrants further investigation.

**Fig. 6:**
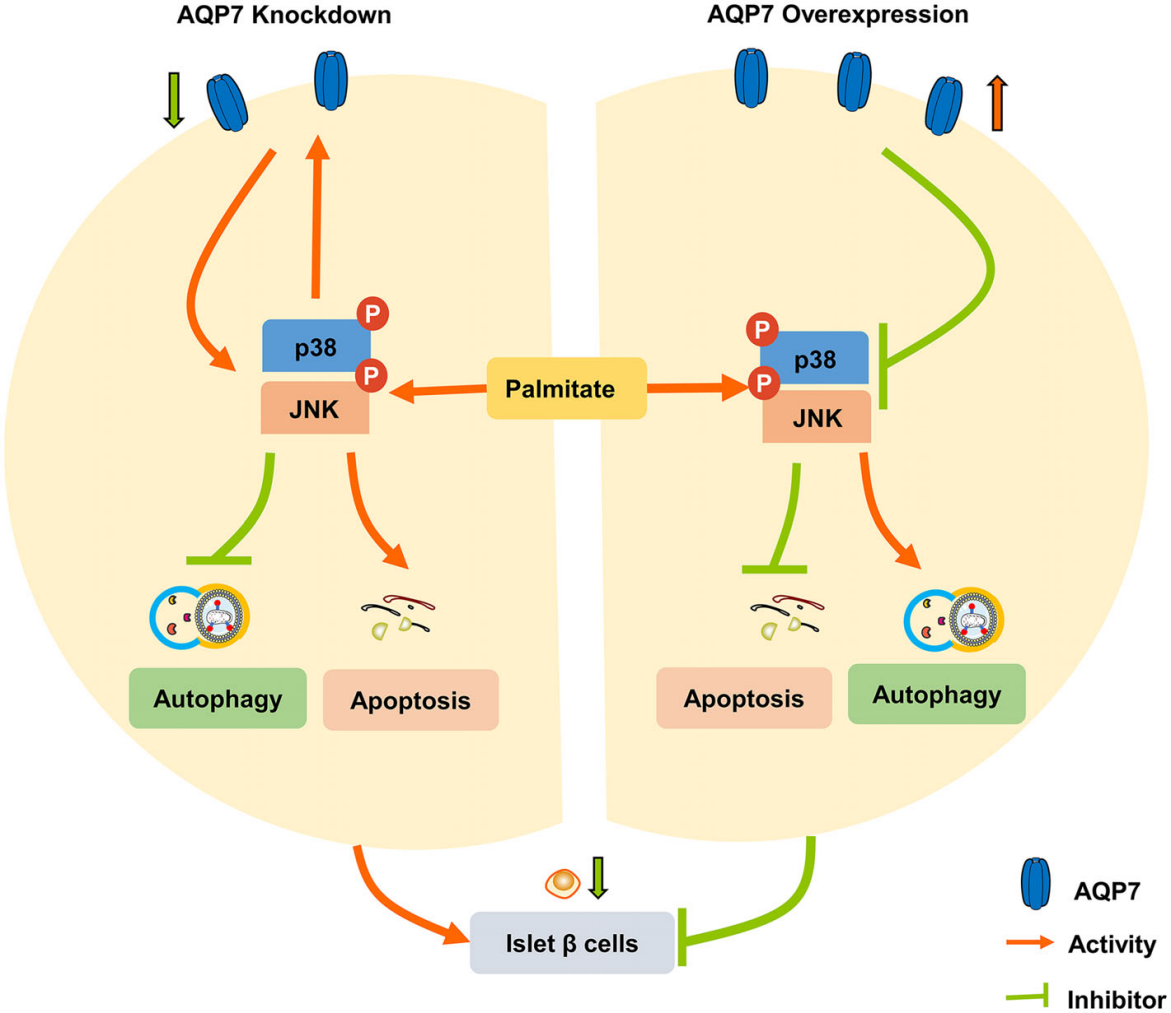
Mechanism of the MAPK and AQP7 positive feedback loop regulating autophagy and apoptosis under lipotoxicity‐challenge. Palmitate (or knockdown AQP7) activates p38 and JNK MAPK signaling pathways, thereby suppressing AQP7 expression, which in turn promotes apoptosis and inhibits autophagy in RIN‐m5f cells. In contrast, AQP7 overexpression inhibited PA‐induced apoptosis and rescued PA‐inhibited autophagy by suppressing the p38 and JNK MAPK signaling pathways.

In summary, our study delineates a mechanism whereby AQP7 and MAPK signaling regulate autophagy and apoptosis in RIN‐m5f cells in the presence of palmitate. Palmitate treatment suppressed AQP7 expression, promoted apoptosis, and inhibited autophagy in RIN‐m5f cells. Notably, the restoration of autophagy and suppression of apoptosis was closely associated with the upregulation of AQP7 expression. Our results also revealed that the downregulation of AQP7 led to an increase in apoptosis, and the activation of the p38 and JNK MAPK signaling pathways. Conversely, overexpression of AQP7 hindered PA‐induced apoptosis and restored autophagy by suppressing the p38 and JNK MAPK signaling pathways. These findings provide a novel perspective on the positive feedback loop between MAPKs signaling and AQP7 in regulating autophagy and apoptosis (Fig. 6), with potential implications for therapeutic interventions in T2DM.

## Conflict of interest

The authors declare no conflict of interest.

## Peer review

The peer review history for this article is available at https://www.webofscience.com/api/gateway/wos/peer‐review/10.1002/2211‐5463.70011.

## Author contributions

MW was responsible for conceptualization, methodology, validation, writing‐original draft. JT was responsible for conceptualization, methodology, writing‐original draft. XH was responsible for data curation. YC was responsible for writing‐review and editing, supervision. MY was responsible for writing‐review and editing, funding acquisition, and conceptualization. GQ was responsible for writing‐review and editing, conceptualization. All authors reviewed the results and approved the final version of the manuscript submitted for publication. All authors reviewed the results and approved the final version of the manuscript submitted for publication.

## Supporting information


**Fig. S1.** Effects of palmitate, p38, and JNK MAPK signaling pathways on apoptosis in RIN‐m5f cells, detected via flow cytometry and Hoechst staining.
