# Supplementary figures and images for: Positive feedback loop between MAPK and aquaporin 7 regulates autophagy and apoptosis induced by palmitate in RIN‐m5f cells
Source: FEBS Open Bio. 2025 Mar 24;15(6):972–84. doi: 10.1002/2211-5463.70011 (PMC12127872; doi:10.1002/2211-5463.70011)

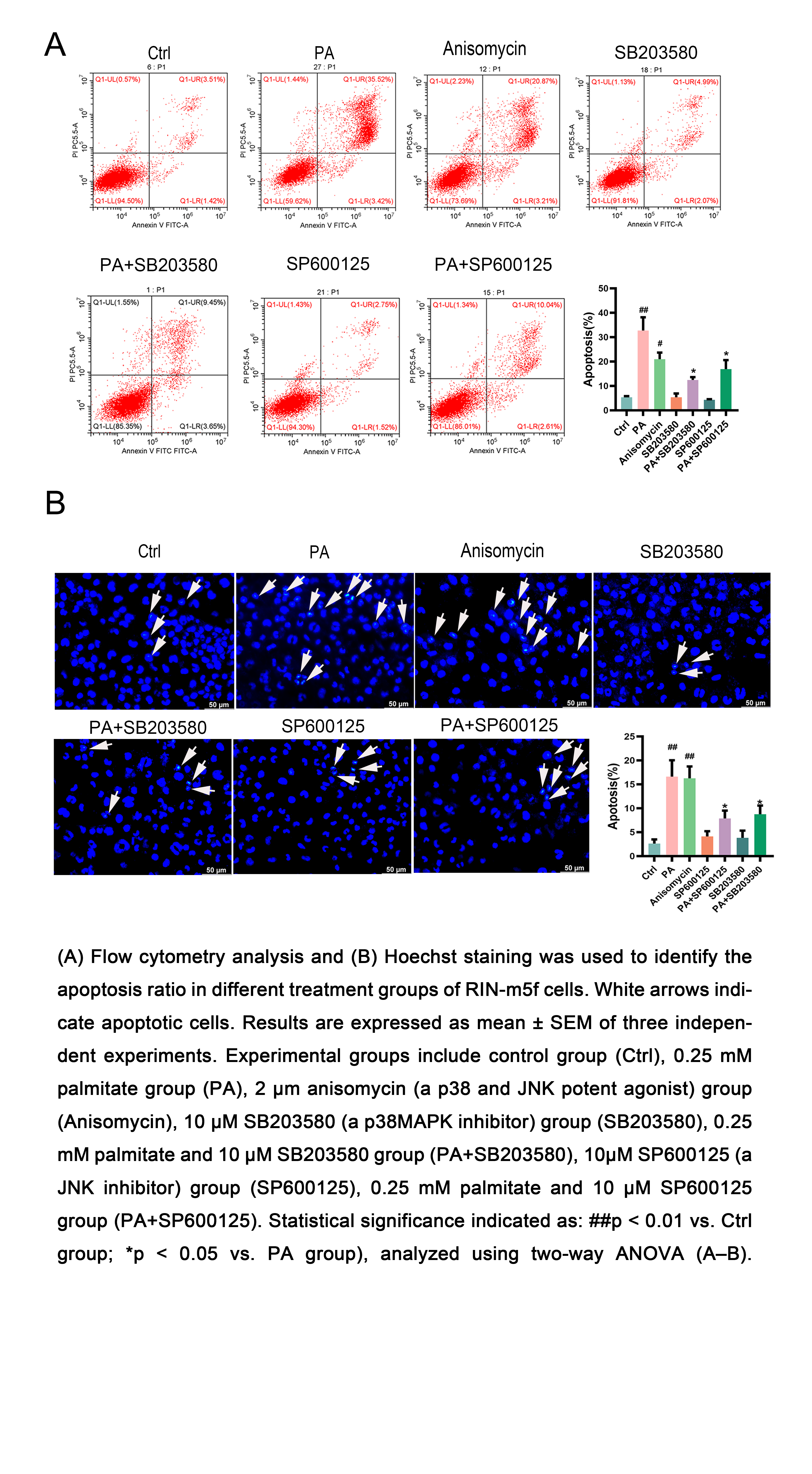

Supplement: Supplementary file 1 — Fig. S1. Effects of palmitate, p38, and JNK MAPK signaling pathways on apoptosis in RIN‐m5f cells, detected via flow cytometry and Hoechst staining. [file FEB4-15-972-s001.tif]
